# Typhlitis: A Rare Appendicitis Mimic in a Young Healthy Female

**DOI:** 10.7759/cureus.15839

**Published:** 2021-06-22

**Authors:** Jonathan Vincent M Reyes, Salman B Syed, Tasur Seen, Nirali Sheth, Christopher Kowalczyk

**Affiliations:** 1 Internal Medicine, Icahn School of Medicine at Mount Sinai, Elmhurst Hospital Center, Elmhurst, USA; 2 Internal Medicine, University of Illinois College of Medicine, Peoria, USA

**Keywords:** typhilitis, neutropenic colitis, enterocolitis, c. diff colitis, appendicitis, right lower quadrant pain, rlq pain

## Abstract

Typhlitis, also known as neutropenic enterocolitis, is a rare but serious condition characterized by inflammation of the cecum causing right lower quadrant (RLQ) pain and fever. It typically affects immunocompromised patients with neutropenia, hematologic malignancies, AIDS, or those on immunosuppressive therapy. This is an entity that should be considered in any differential for a patient with febrile RLQ pain, not just those with obvious immunosuppression.

## Introduction

Typhlitis, a severe inflammatory state of the cecum is a rare condition affecting mainly neutropenic and immunosuppressed patients. It typically affects immunocompromised patients with neutropenia, hematologic malignancies, AIDS, or those on immunosuppressive therapy [1]. It frequently mimics appendicitis on presentation with a triad of abdominal pain, fever, and neutropenia, and it is diagnosed using computed tomography (CT) imaging. However, given the extent of morbidity and mortality, early diagnosis for immunocompromised patients is imperative. There is limited literature on the incidence and pathogenesis of typhlitis as well as few reported cases on non-immunocompromised patients [2].

## Case presentation

A 25-year-old female, reporting no past medical history, presents to the emergency department with a one-week history of recurrent right lower quadrant (RLQ) abdominal pain. She describes three episodes of non-bloody diarrhea in the morning. She denies fevers, nausea, vomiting, or prandial/postprandial pain. She has no personal or family history of cancer or autoimmune diseases.

**Figure 1:**
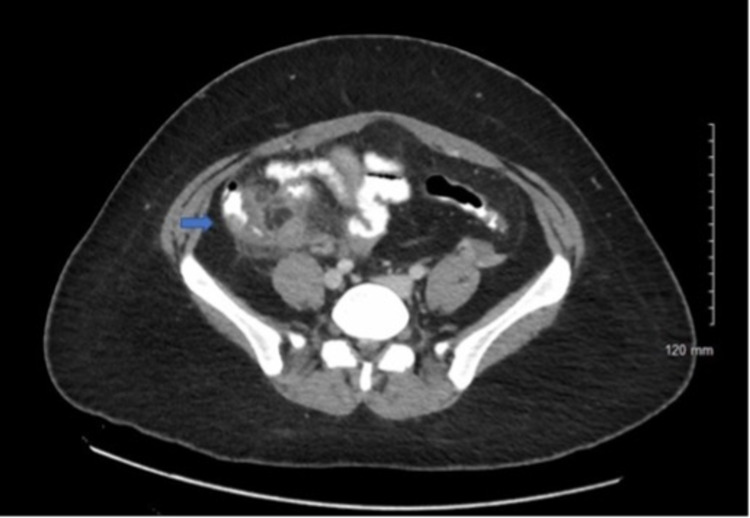
CTAP with contrast: transverse view demonstrating inflammatory thickening of cecum CTAP, Computed tomography arterial portography.

**Figure 2:**
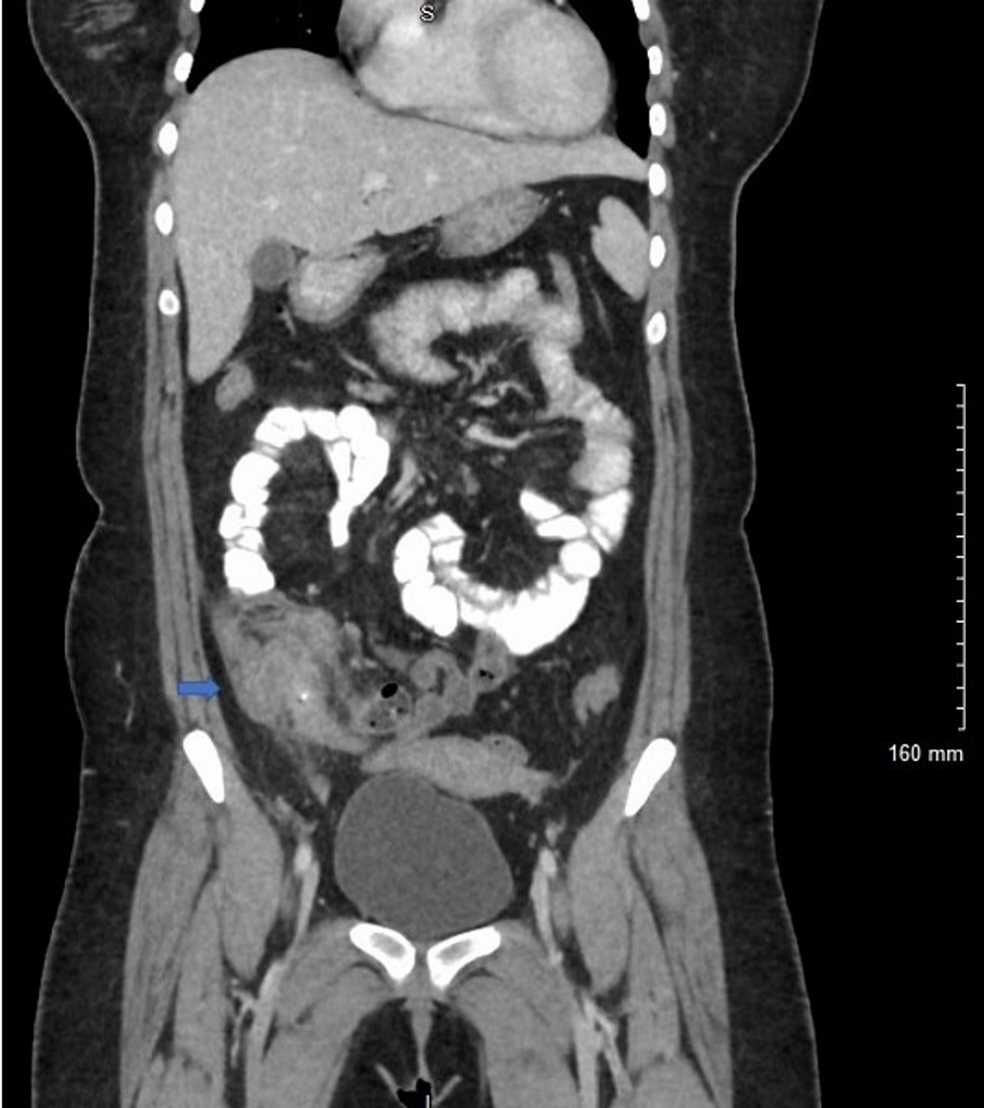
CTAP with contrast: coronal view demonstrating inflammatory thickening of cecum CTAP, Computed tomography arterial portography.

On physical exam, vitals are significant for fever to 100.9°F. The abdomen is soft, mildly distended, moderately tender to palpation in the RLQ, and without rebound tenderness. Initial lab work was remarkable for microcytic anemia with hemoglobin (Hgb) to 10, mean corpuscular volume (MCV) 76, leukocytosis to 12.32, absolute neutrophil count 1196 (2.10-7.60 x 10^3^ cells/μL), lactate 1.1, and HIV-negative. The clinical picture was concerning for appendicitis. CT abdomen and pelvis demonstrated inflammatory thickening of the cecum (Figures 1, 2). The patient was admitted to general surgery for conservative management with bowel rest, intravenous (IV) Zosyn, and pain management. The patient improved and was discharged home on oral antibiotics. During her primary care follow-up visit, the patient endorsed the resolution of her symptoms.

## Discussion

Neutropenic enterocolitis, more commonly referred to as typhlitis, is a severe inflammatory disorder of the intestines that generally occurs in neutropenic and immunosuppressed patients. Given the described population, it may not be surprising that morbidity in this pathology is estimated between 21% and 48% [3].

Conventionally, it is thought that patients cannot have typhlitis without lab-confirmed immunosuppression [4]. However, the diagnosis is confirmed with radiologic evidence. While the pathogenesis is not well understood, it is thought that neutropenia and states of immunosuppression may lead to intestinal edema and disrupted mucosal surface. This disruption allows for the translocation of bacteria. Treatment consists of IV antibiotics and bowel rest. Surgery is considered for complications related to necrosis [5,6].

Our patient had a typical presentation and compatible CT findings; notably, identification of cecitis despite leukocytosis led to conservative versus operative management as well as diagnosis of typhlitis. Ultrasound is typically the first radiologic evaluation for this presentation as it is a faster modality with high sensitivity for appendicitis and typhlitis [7], but considering further characterization of bowel inflammation on CT has been recommended when it does not significantly delay diagnosis. Bowel wall thickening and inflammation of the cecum detected by CT have been proposed as the main criterion to establish the diagnosis of typhlitis [2,4,5,7-9]. The extent of bowel wall thickening is also a valuable prognostic factor, which adversely affects the outcome [9]. In addition to these findings, appendiceal dilatation on CT is typically observed with appendicitis, which was not visualized in our patient.

When considering the same presentation as above but with a different etiology, *Clostridium difficile* (*C. diff*) colitis is another severe disease on the differential in this patient population. Between these three pathologies (appendicitis, typhlitis, *C. diff* colitis), there is a great overlap in the symptomatology and patient history, so radiologic variables become increasingly important for narrowing the diagnosis. Given that some degree of bowel necrosis is common in typhlitis, pneumatosis intestinalis on CT imaging is most associated with typhlitis. Comparably, bowel wall thickening is non-specific and can be found in all three disease states, but wall nodularity is highly specific for *C. diff* colitis [8].

## Conclusions

In conclusion, this case is to inform clinicians of a rare appendicitis mimic. This case aims to broaden the possible differential diagnosis for patients who present with nausea, vomiting, fever, and RLQ pain. Imaging will assist with an early diagnosis, which will in turn limit complications and potentially reduce morbidity. Further research is warranted to see how early diagnosis affects patient outcomes.
